# A Ten-MicroRNA Signature Identified from a Genome-Wide MicroRNA Expression Profiling in Human Epithelial Ovarian Cancer

**DOI:** 10.1371/journal.pone.0096472

**Published:** 2014-05-09

**Authors:** Lin Wang, Miao-Jun Zhu, Ai-Min Ren, Hong-Fei Wu, Wu-Mei Han, Ruo-Ying Tan, Rui-Qin Tu

**Affiliations:** 1 Department of Obstetrics and Gynecology, Shanghai Zhongshan Hospital, Fudan University, Shanghai, China; 2 Biovue Technology (China) Ltd., Shanghai, China; Sun Yat-sen University, China

## Abstract

Epithelial ovarian cancer (EOC) is the most common gynecologic malignancy. To identify the micro-ribonucleic acids (miRNAs) expression profile in EOC tissues that may serve as a novel diagnostic biomarker for EOC detection, the expression of 1722 miRNAs from 15 normal ovarian tissue samples and 48 ovarian cancer samples was profiled by using a quantitative real-time polymerase chain reaction (qRT-PCR) assay. A ten-microRNA signature (hsa-miR-1271-5p, hsa-miR-574-3p, hsa-miR-182-5p, hsa-miR-183-5p, hsa-miR-96-5p, hsa-miR-15b-5p, hsa-miR-182-3p, hsa-miR-141-5p, hsa-miR-130b-5p, and hsa-miR-135b-3p) was identified to be able to distinguish human ovarian cancer tissues from normal tissues with 97% sensitivity and 92% specificity. Two miRNA clusters of miR183-96-183 (miR-96-5p, and miR-182, miR183) and miR200 (miR-141-5p, miR200a, b, c and miR429) are significantly up-regulated in ovarian cancer tissue samples compared to those of normal tissue samples, suggesting theses miRNAs may be involved in ovarian cancer development.

## Introduction

Ovarian Cancer (OC), one of the three gynecologic malignancies, is the seventh most common cancer among women worldwide [1]. Inheritance of high-penetrance cancer susceptibility genes such as mutated BRCA1 or BRCA2 and/or Lynch syndrome-associated mutations pose an increased risk of developing OC [2]. Epithelial Ovarian Cancer (EOC) accounts for about 80–90% of OCs [3]. EOC is the most lethal gynecologic malignancy in Western countries [4]. In the United States of America (USA), EOC caused almost 15,500 deaths in 2012 [5]. There are only a few effective biomarkers and therapies for EOC [6]–[8], and EOC's early detection still remains a challenge for oncologists. The 5-year survival rate of more than 70% of patients with advanced-stage EOC is only 35% [5]. No effective screening method to detect early-stage OC with high specificity and sensitivity is currently available, and cancer antigen-125 together with transvaginal ultrasonography can detect only 30–45% of patients with early-stage disease [9]. Albeit deoxyribonucleic acid (DNA) methylation biomarkers play a promising role in detecting EOC, there is still a huge need to identify potential biomarkers with high specificity and sensitivity. The analytical techniques also need to be standardized in order to improve detection, optimize treatment, and achieve desirable patient outcomes [10].

The role of micro-ribonucleic acids (miRNAs) in OC has gained recent attention, since they offer novel strategies for prevention, early detection, diagnosis, and treatment. They play important roles in essential processes such as cell differentiation, growth, and apoptosis [11], [12]. Aberrant expression or mutation of miRNAs in cancers indicated their potential to act as a novel class of oncogenes or tumor suppressor genes based on their targets [13]. Since miRNAs could be isolated and detected from tissue and blood samples, peripheral blood-derived miRNAs were used as novel circulating biomarkers for OC [14]. In most published studies, miRNA expression was profiled by miRNA microarray and confirmed by quantitative polymerase chain reaction (qPCR).

The present study conducted a genome-wide tissue miRNA expression profiling by quantitative real-time PCR reaction. A profile of 10 tissue miRNAs was found, which may serve as a biomarker for EOC and contribute to better understanding of the mechanism of ovarian tumor genesis and development.

## Materials and Methods

### Collection of OC tissue samples

Normal epithelial ovarian tissue samples (N samples) (n = 15) and malignant epithelial ovarian tissue samples (C samples) (n = 48) were collected at Zhongshan Hospital of Fudan University, Shanghai, China. The 48 malignant epithelial ovarian tissue samples (C samples) included 41 epithelial ovarian carcinoma samples (CE samples) and 7 epithelial ovarian cancer borderline tissue samples (CB samples). The 48 malignant epithelial ovarian tissue samples were of different cell types: 29 serous, 6 mixed epithelial, 6 endometrioid, 1 adenocarcinoma (not otherwise specified), 4 clear cells, and 2 mucinous carcinomas. All tissue samples were immediately frozen in liquid nitrogen after being removed from body and stored at −80°C for long term storage. Written informed consents were obtained from all subjects, and the study protocol was approved by the Ethics Committee of Zhongshan Hospital. The demographics and clinical features of the patients and normal controls are listed in Table 1.

**Table 1:** Summarized characteristics of tissue samples.

Characteristics	OC samples from epithelial cells	OC samples from OC borderline tissue	Control samples from epithelial cells
	(*n* = 41)	(*n* = 7)	(*n* = 15)
Median age at diagnosis (y)	57	57	56
**Tumor histology**			
** Serous**	**28**	**1**	
** Mixed epithelial**	**1**	**5**	
** Endometroid**	**6**	**0**	
** Adenocarcinoma NOS**	**1**	**0**	
** Clear cell**	**4**	**0**	
** Undifferentiated**	**1**	**1**	
**FIGO stage**			
** I**	**7**	**7**	
** II**	**5**	**0**	
** III**	**27**	**0**	
** IV**	**2**	**0**	
**Tumor grade**			
** 1**	**8**	**0**	
** 2**	**11**	**0**	
** 3**	**17**	**1**	
** Undetermined**	**5**	**6**	

OC =  Ovarian Cancer.

y =  years.

FIGO =  the TNM and International Federation of Gynecology and Obstetrics.

Adenocarcinoma NOS =  adenocarcinoma not otherwise specified.

### miRNA isolation

Total RNA was isolated from <50 mg of frozen tissue with miRNeasy Mini Kit (*Qiagen, USA*) per manufacturer's instructions. The quality of the isolated RNA was detected by agarose gel electrophoresis, and the quantity was analyzed by an ultraviolet spectrophotometric method using Biomate3 (*Thermo Scientific, USA*). The total RNAs with sharp bands of 18S rRNA and 28S rRNA are considered non-degraded and used for miRNA profiling.

### Addition of Poly(A) tails and reverse transcription

The purified total RNA (including small RNA) was diluted to 125 ng/µl with 0.1x RNA storage buffer (Ambion, USA) containing 0.1% Tween-20 (*Sigma*). miRNAs were added a poly(A) tail and reverse transcripted into cDNA using Sharpvue miRNA First Strand Kit (*Biovue, Shanghai, China*) per the instructions in the kit. The concentration of total RNA in the reaction of addition of poly(A) tails and reverse transcription is 50 ng/µl.

### Real-time PCR

The synthesized miRNA cDNA was mixed with Sharpvue 2x Universal qPCR Master Mix (*Biovue, Shanghai, China*) and nuclease-free water. The Sharpvue Human miRNA Primer Array-A-E v1.0 384-well (*Biovue, Shanghai, China*) was used, and the real-time PCR was performed per the instructions in the Sharpvue miRNA qPCR Kit. Each sample was detected by 1757 miRNA primers including 35 controls in five 384-well plates. Each plate contains three endogenous controls (hsa-7SL-scRNA, hsa-RNU6B, and hsa-RNU48) in duplicate and one no template control. miRNAs expression levels were quantified using ABI 7900HT Fast Real-Time PCR System (*Applied Biosystems, USA*). The real-time PCR reaction was incubated at 95°C for 2 minutes, followed by 3 cycles of 96°C for 5 seconds and 60°C for 1 minute, 37 cycles of 96°C for 5 seconds and 60°C for 30 seconds, and running melting curve at the end. EVA-Green dye and Rox dye were used as reporter and reference, respectively. Details of miRNA detection are shown in the Figure S1 in file S1.

### Statistical analysis

Data analysis was performed using R and Bioconductor packages. Of the 1722 miRNA assays and 2 endogenous control assays (hsa-RNU6B, and hsa-RNU48), 1696 miRNAs had Ct value below Ct = 32 (detection threshold) among at least 10% of the detected 63 samples. The remaining 28 assays were removed from further analysis. In order to remove differences in sample RNA input, Quantile-Median method was used to process the raw Ct measurements [15]. Samples that showed significant difference in profiles (mean absolute difference, Bioconductor package “arrayQualityMetrics”) were considered as outliers and were removed from downstream analysis. This procedure removed three OC tissues (two epithelial carcinoma tissue samples and one epithelial ovarian cancer borderline tissue sample) and one normal epithelial ovarian tissue sample.

Differential expression analysis was performed on the remaining 59 samples using t-test (R package “limma”). miRNAs producing false discovery rate (FDR)-adjusted p-values below 0.1 and fold change above 2 were called differentially expressed.

In order to develop a prediction algorithm for OC diagnosis from a population of samples containing 39 epithelial ovarian carcinoma tissues together with 6 epithelial ovarian cancer borderline tissues and 14 normal epithelial ovarian tissues, three classification methods were tested: support vector machine (SVM, Bioconductor package “e1071”), K-nearest neighbors (Bioconductor package “class”), and diagonal linear discriminant analysis (Bioconductor package “sfsmisc”). The performance of algorithms was initially evaluated using leave-one-out cross validation procedure for different number of predictor markers. For each set of training samples, miRNAs were ranked based on their t-test p-value generated when comparing cancer tissues against normal tissues. The top n miRNAs (where n is allowed to range between 2 and 50) were used to build a prediction model based on the information on training samples and applied to the remaining test sample. Prediction class and probability were recorded for every sample and classification algorithm. Stability of the miRNA predictor lists used with training samples was evaluated by the percent overlap of the top n miRNAs of these lists. Because of the limited number of samples available for this study, the common miRNA of these lists were chosen as the final list of predictors (selected markers). Significant differences were determined using the Student's t-test and considered to be significant if P value<0.05. In the analysis, sensitivity is defined as the percentage of cancer tissues that are correctly identified as having this condition, while specificity is defined as the percentage of normal tissues that are correctly identified as normal.

## Results

### Clinical and Pathological findings

The clinical features of the 45 patients with OC and 14 normal controls used in our study are summarized in Table 1. Ages of patients with OC ranged from 20 to 81 years (median, 57 years) while normal controls ranged between 21 and 75 years (median, 56). All these samples were diagnosed by pathology and had macroscopic description. Per the 2012 Soft Tissue OC Guideline of National Comprehensive Cancer Network, the different tumor differentiation grades of patients in this study included the following: 8 cases with grade I (16.7%), 11 cases with grade II (23%), 18 cases with grade III (37.5%), and 11 cases with undetermined (23%); and the tumor stages were 14 cases with stage I (29.2%), 5 cases with stage II (10.4%), 27 cases with stage III (56.3%), and 2 cases with stage IV (4.2%) cancers.

### miRNA expression comparison

In order to remove differences in RNA inputs used to profile the 63 ovarian tissue samples in this study, the Quantile-Median method [16] was used to process the raw Ct measurements. Overall, the data of 59 ovarian tissue samples were analyzed for the miRNA expression.

**Figure 1:**
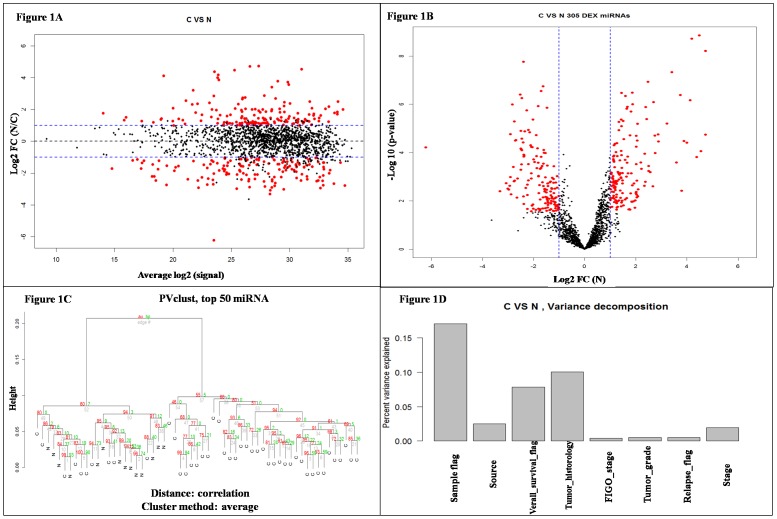
Comparison between 45 ovarian cancer (39 epithelial carcinoma and 6 borderline tissues) and 14 normal ovarian tissues. A) The plot of miRNA assays used to profile compared samples: fold change (y-axis) against normalized Ct measurements. B) Volcano plot of the resulting p-values of the t-test (y-axis) between the C and N groups. 305 miRNAs show FDR-adjusted p-values below 0.1 and fold change above 2 (shown in red). C) Hierarchical clustering (R package pvclust) of the 45 ovarian cancer tissues and 14 ovarian normal tissues based on top 50 most variable miRNA assays. For each cluster in hierarchical clustering, quantities called *p*-values (approximately unbiased *p*-value (red) and Bootstrap Probability p-value (green)) are calculated via multi-scale bootstrap resampling. *P*-value of a cluster is a value between 0 and 1, which indicates how strong the cluster is supported by data. D) 17% of the variance observed in the Ct measurements of top 50 most variable miRNA assays across all samples can be explained by sample pathology status (C or N). The remaining covariates considered here (source = hospital source, survival, tumor histology, FIGO stage, tumor grade, relapse, and stage) explain 24% of the variance.

To investigate the ability to identify a miRNA expression signature of OC from tissue samples, miRNA expression profiles of 59 ovarian tissue RNA samples collected from 39 epithelial cancer tissue samples, 6 epithelial ovarian cancer borderline tissue samples, and 14 normal epithelial ovarian tissue samples were analyzed using the 1696 miRNA assays detected (Ct<32) in at least 10% of the samples in this study. The 45 OC tissues were compared to the 14 normal ovarian tissues using t-test. 305 miRNAs were found to have FDR-adjusted p-value below 0.1 and fold change above 2. A summary of these findings are presented in Figure 1. Differentially-expressed miRNAs spanned a large range of Ct values and fold changes.

### Comparison of miRNA expression between different types of EOC tissue samples

The miRNA expression of the 59 tissue samples that were compared included 45 samples comprising the C group [including 39 epithelial ovarian cancer tissue samples (CE group) and 6 epithelial ovarian cancer borderline tissue samples (CB group)] and 14 normal epithelial ovarian tissue samples (N group).

The hierarchical clustering of the miRNAs was shown by FDR-adjusted p-values below 0.1 and fold-change above 2 (red point). The comparisons for 335 differentially-expressed miRNAs in the CE and N groups are shown in the Figure S2 in file S1. In contrast, no candidate miRNAs were observed when the CB group was compared with the N group (Figure S3 in file S1) and when the CE group was compared with the CB group (Figure S4 in file S1). The reduced number of cancer borderline tissues available for this study could limit the power in detecting significant changes and explain the lack of changes observed in these comparisons.

### Biomarker selection

**Figure 2:**
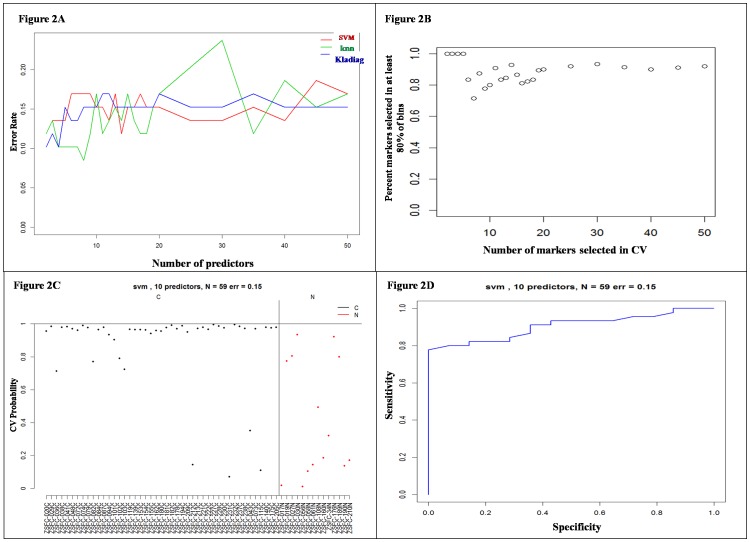
Determination of error rates by leave-one-out cross validation vs. number of markers and miRNA markers overlapping. A) Error rate produced by different classification algorithms as a function of the number of prediction markers used. Leave-one-out cross-validation procedure was used to estimate resulting error rates. B) Percent overlapping of predictor miRNA selected from the different training sets of samples used. C) Leave-one-out cross validation results: each sample class probability (y-axis) is estimated based on SVM model learned from all other samples. Tissues (cancer black, normal red) are represented by classification probability of being cancer. D) ROC curve based on leave-one-out cross validation results using SVM method.

Accuracy of the three tested methods for different number of predictor markers ranging between 2 and 50 is shown in Figure 2. Prediction accuracy was relatively similar between the methods used, and a better performance for classification models was based on reduced number of miRNA markers (Figure 2A). Stability (y-axis) measured as percent overlap between the lists of miRNA markers used for classification (59 in this analysis, one for each sample used for testing) is presented in Figure 2B as a function of number of markers used (x-axis). As expected, the overlap between the selected lists of miRNA predictors increased to approximately 90% when prediction was based on 11 or more markers, suggesting a reduced effect in marker selection step from individual samples.

### miRNA screening and testing between the ovarian cancer and normal groups

The 10 common miRNAs across the lists of used markers for predictions based on 11 miRNAs were selected as final list of biomarkers for OC diagnosis. Some characteristics of these miRNAs, including fold change between cancer and normal tissues together with the t-test p-values and FDR-adjusted p-values, are presented in Table 2. The 10 miRNAs included miR-1271-5p and miR-574-3p, which had significantly lower expression (P values are presented in Table 2) levels in the ovarian cancer group (C group) than in the normal group. In contrast, other miRNAs such as miR-182-5p, miR-183-5p, miR-96-5p, miR-15b-5p, miR-182-3p, miR-141-5p, miR-130b-5p, and miR-135b-3p had a significantly higher expression level in ovarian cancer tissue sample group (C group) than in the normal group (P values are presented in Table 2).

**Table 2:** Selected list of biomarkers for ovarian cancer diagnosis.

Assay_Name	miRID	logFC	AveExpr	t	P.Value	adj.P.Val
hsa-miR-182	hsa-miR-182-5p	4.247	23.814	7.14	1.33E-09	1.44E-06
hsa-miR-183	hsa-miR-183-5p	4.517	25.219	7.078	1.70E-09	1.44E-06
hsa-miR-96	hsa-miR-96-5p	4.759	26.649	6.765	5.86E-09	3.31E-06
hsa-miR-1271	hsa-miR-1271-5p	−2.44	25.811	−6.536	1.44E-08	6.11E-06
hsa-miR-182#	hsa-miR-182-3p	3.402	28.455	6.18	5.79E-08	1.97E-05
hsa-miR-574-3p	hsa-miR-574-3p	−1.636	20.244	−5.998	1.17E-07	3.32E-05
hsa-miR-141#	hsa-miR-141-5p	2.454	29.51	5.955	1.39E-07	3.37E-05
hsa-miR-130b#	hsa-miR-130b-5p	1.905	27.94	5.858	2.02E-07	4.27E-05
hsa-miR-15b	hsa-miR-15b-5p	1.452	18.971	5.787	2.66E-07	4.63E-05
hsa-miR-135b#	hsa-miR-135b-3p	3.721	29.773	5.78	2.73E-07	4.63E-05

miRID = miRID from miRBase version 20.

Log FC =  log fold change.

Ave. Expr =  average expression.

Adj. P. Val =  Adjustment of P Value.

**Figure 3:**
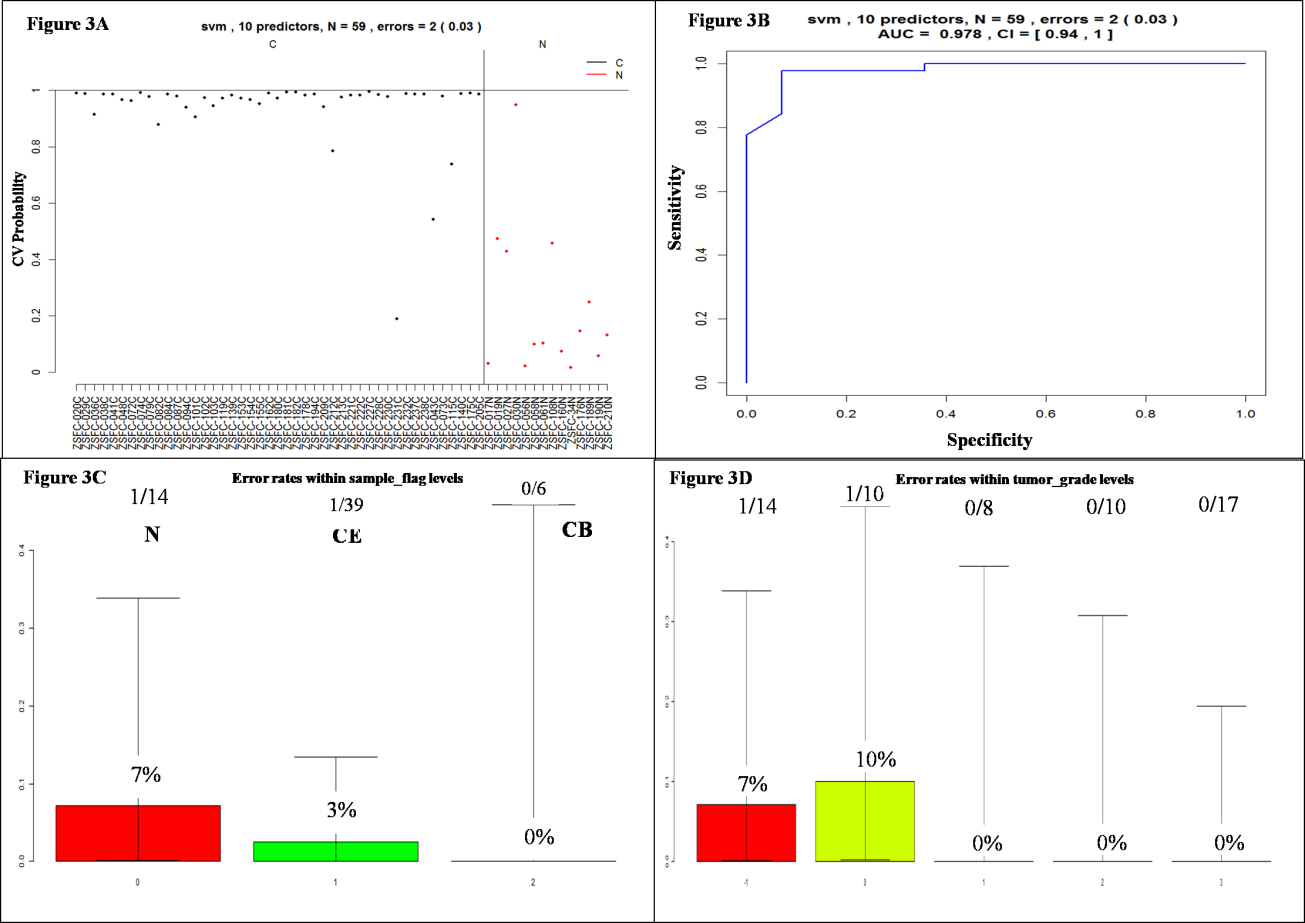
Ovarian cancer classification performance for the 10 selected miRNAs (**Table 2**) using SVM algorithm and leave-one-out cross-validation. A) Prediction probabilities (ovarian cancer) for each sample used in this study (C = Cancer; N = Normal). B) ROC curve. C) Prediction error across different tissue groups: normal tissue (N), epithelial carcinomas tissues (CE) and borderline tissues (CB). D) Prediction error within tumor grade groups [Increased error in lower grade samples (but not significant)].

The classification performance of the selected 10 miRNAs for SVM algorithm after the use of leave-one-out cross-validation procedure is presented in Figure 3. Since the list of selected miRNA used all samples, the performance presented here might be an overestimate of the true one. This was reflected in the improved accuracy of prediction performance when marker selection step was independent on the testing set of samples (Figure 3). The observed accuracy was 86%, while the observed accuracy for selected markers was 96%. The sensitivity in detecting OC based on selected markers was 97%, while the specificity was 92% with an area under the curve (AUC) of the resulting receiver operating characteristic (ROC) curve of 0.978. No significant differences were observed across the major clinical factors collected for samples tested in this study (Figure 3D).

## Discussion

In order to find specific profiles of tissue-derived miRNAs of EOC, a comparative study was performed using a qRT-PCR array platform between 45 epithelial cells tissue samples from patients with EOC and 14 epithelial cells normal tissue samples from healthy controls. The study revealed that 10 dysregulated miRNA signature among which hsa-miR-1271-5p and hsa-miR-574-3p were down-regulated; and hsa-miR-182-5p, hsa-miR-183-5p, hsa-miR-96-5p, hsa-miR-182-3p, hsa-miR-141-5p, hsa-miR-15b-5p, hsa-miR-130b-5p, and hsa-miR-135b-3p were overexpressed in ovarian cancer tissues. These findings could effectively distinguish OC tissues from ovarian normal tissues, implying the presence of specific miRNAs in EOC. The 10-miRNA signature identified in the study may contribute to better understanding of the mechanism of EOC tumorigenesis and development and also help in the diagnosis of EOC.

miRNA expression system used in the study possessed the following significant advantages: (1) the largest collection of validated assays (1722 human miRNAs in miRBase v 20.0), (2) one of the largest detection range (up to 6 orders of magnitude), (3) the most sensitive detection (down to a single copy number), (4) the highest specificity (<3% cross-reactivity among 8 members of let-7 family). Three different classification methods were used with a leave-one-out cross validation procedure to minimize errors. To the best of our knowledge, this is the largest scale of miRNAs (1722) expression profiling study conducted using qRT-PCR so far.

We had analyzed the variance decomposition of cancer and normal tissue samples and showed the result in figure 1D. We recognized that there is no correlation with the hospital source, survival, the tumor histology, the disease stage, the tumor grade and even the relapse, except the samples (normal tissue samples vs. malignant tissue samples). More importantly, the 10 miRNAs discriminated the EOC group from normal group with high sensitivity and specificity, suggesting their potential value for early detection of OC. They indicated that the classification of C group from N group could be expected to have 97% sensitivity and 92% specificity.

The 10-miRNA signature identified in the study showed prominent differentiation between EOC from normal controls. Among these miRNAs, miR-96, miR-182 and miR-183 are clustered at one locus of the chromosome 7 [17] and miR-141-5p belongs to the miR-200 family, which is clustered on the chromosomes 12. Not just miR-141, we further found that other members (200a/b/c) of miR200 family are also overexpressed in EOC comparing to the normal (Table 3). Both two miRNA clusters are well-known oncogenic miRNA clusters that have been extensively reported to involve in tumor genesis in many types of tumors [18]–[20]. The miR-200 family members were reported to target ZEB1 and ZEB2, ZEB transcription factors are crucial repressors of BMP signaling, and Peart et al reported that BMP signaling controls the malignant potential of ascites-derived human epithelial ovarian cancer spheroids via AKT kinase activation [21]. The members (miR200b and miR429) of miR200 family were reported to be significantly associated with ovarian cancer recurrence and overall survival [18]. For miR183-96-182 cluster, Xu reported that overexpressed miR-182 and miR-96 targeting fork head box O3 plays a significant role in the pro-proliferation effect of leptin on ovarian cancer cells [22]. miR-182 was reported to directly and negatively regulates an important tumor suppressor, programmed cell death 4 (PDCD4) in OC [23]. miR-183 is confirmed to have negative regulatory effects on Tiam1 expression, which contributes to the invasive, migratory, and viability properties of OC cells [24]. A recent study by sequencing small RNAs from isogenic p21+/+ and p21−/− cells demonstrated that the several miRNA clusters, miR-200b-200a-429, miR-200c-141 and miR-183-96-182 were down-regulated in p21-deficient cells, if adding antagonizing miR-200 and miR-183-96-182 cluster miRNAs in p21+/+ cells, it increased invasion and elevated the levels of *VIM*, *ZEB1* and *SLUG* mRNAs, which are common targets of miR-183 and miR-96. The study further found that p21 forms a complex with ZEB1 at the miR-183-96-182 cluster promoter to inhibit transcriptional repression of this cluster by ZEB1 suggesting a reciprocal feedback loop [25]. Some studies have found that miR-200 family members were up-regulated in serous epithelial ovarian cancer cell line as well as in serum from patients with serous epithelial ovarian cancer [26], [27].

**Table 3:** miRNA expression profile of miR-183-96-182 cluster and miR200 cluster in ovarian cancer.

Assay_Name	miRID	Precursor Chromosome Location	Cluster Name	LogFC (CE+CB vs Normal)	LogFC (CE vs. Normal)
hsa-miR-183	hsa-miR-183-5p	chr7:129414745-129414854 [-]	miR183-96-182	4.52	4.72
hsa-miR-183#	hsa-miR-183-3p	chr7:129414745-129414854 [-]	miR183-96-182	2.36	2.41
hsa-miR-96	hsa-miR-96-5p	chr7:129414532-129414609 [-]	miR183-96-182	4.76	4.86
hsa-miR-182	hsa-miR-182-5p	chr7:129410223-129410332 [-]	miR183-96-182	4.25	4.40
hsa-miR-182#	hsa-miR-182-3p	chr7:129410223-129410332 [-]	miR183-96-182	3.40	3.48
hsa-miR-200a#	hsa-miR-200a-5p	chr1:1103243-1103332 [+]	miR200a-200b-429	1.70	1.78
hsa-miR-200a	hsa-miR-200a-3p	chr1:1103243-1103332 [+]	miR200a-200b-429	4.02	4.06
hsa-miR-200b#	hsa-miR-200b-5p	chr1:1102484-1102578 [+]	miR200a-200b-429	2.74	2.78
hsa-miR-429	hsa-miR-429	chr1:1104385-1104467 [+]	miR200a-200b-429	4.39	4.58
hsa-miR-200c	hsa-miR-200c-3p	chr12:7072862-7072929 [+]	miR200c-141	4.12	4.35
hsa-miR-200c#	hsa-miR-200c-5p	chr12:7072862-7072929 [+]	miR200c-141	2.69	2.74
hsa-miR-141	hsa-miR-141-3p	chr12:7073260-7073354 [+]	miR200c-141	3.92	4.03
hsa-miR-141#	hsa-miR-141-5p	chr12:7073260-7073354 [+]	miR200c-141	2.45	2.42

miRID = miRID from miRBase version 20.

Log FC (**CE+CB vs. Normal**) = log2 fold change for (**CE+CB vs. Normal**).

Log FC (**CE vs. Normal**) = log2 fold change for (**CE vs. Normal**).

These results suggest that the clusters of the miR-183-96-182 and miR200 play a very important role in EOC and may be used as potential diagnostic biomarkers for EOC detection as well as have therapeutic potential for the suppression of ovarian cancer proliferation and invasion.

The SVM model was further used with profiling data generated from epithelial cell samples as training data to test epithelial ovarian cancer borderline tissue samples. Six out of the 7 OC samples were predicted as OC, which indicated that the identified signature could also be useful to detect OC from borderline tissue samples (data not shown). However, this may need a larger sample size for confirmation. However, no significant miRNAs could separate CB from N group, or CB from CE group. This could be attributed to the reduced power in detecting differences between 39 of epithelial ovarian cancer samples (CE) and 6 epithelial ovarian cancer borderline tissue samples (CB). Further studies with larger sample size are needed to confirm this explanation.

Some important differences in miRNAs were also observed for a separation of epithelial ovarian cancer samples (without the epithelial ovarian cancer borderline tissue samples) from normal samples. The seven dysregulated miRNAs including hsa-miR-182-5p, hsa-miR-183-5p, hsa-miR-96-5p, hsa-miR-1271-5p, hsa-miR-182-3p, hsa-miR-1468-5p, and hsa-miR-135b-3p (Table S1 in file S1) were confirmed by the AUC of ROC curve (AUC = 0.965) with 97% sensitivity and 85% specificity (Figure S5 in file S1). 6 out of these 7 miRNAs (hsa-miR-182-5p, hsa-miR-183-5p, hsa-miR-96-5p, hsa-miR-1271-5p, hsa-miR-182-3p, and hsa-miR-135b-3p) are in the 10-miRNAs signature. The 10 unique miRNA profiles distinguished epithelial ovarian tumor tissue samples including epithelial ovarian cancer tissue samples and epithelial ovarian cancer bound line tissue samples from normal epithelial ovarian tissue samples with 97% sensitivity and 92% specificity. The expression profile of 7 miRNA signature was also able to classify epithelial ovarian cancer tissue samples and normal epithelial ovarian tissues. These findings may provide a basis for future studies on clinical value of tumor miRNAs in predicting therapeutic efficacy, maintaining surveillance, and forecasting prognosis and help better understanding of the mechanism of epithelial ovarian tumor genesis and development.

## Supporting Information

File S1 **Supporting information figures. Figure S1, Overview of the experimental design. Figure S2, Comparison between the ovarian epithelial carcinomas tissue (CE group) and normal tissue (N group).** A) MA plot of assays used to profile compared samples. B) Volcano plot of the resulting p-values of the t-test between the CE and the N groups. 335 miRNAs shows adjusted p-values (FDR) below 0.1 and fold-changes above 2 (shown in red). C) Hierarchical clustering of CE and N groups based on top 50 most variable miRNA assays. **Figure S3**, Comparison between the ovarian borderline tissue (CB group) and normal tissue (N group). A) MA plot of assays used to profile compared samples. B) Volcano plot of the resulting p-values of the t-test between the CB and the N groups. No miRNA shows adjusted p-values (FDR) below 0.1 and fold-changes above 2 (shown in red). C) Hierarchical clustering of CB and N group based on top 50 most variable miRNA assays. **Figure S4, Comparison between the ovarian epithelial carcinomas tissue (CE group) and ovarian borderline tissue (CB group).** A) MA plot of assays used to profile compared samples. B) Volcano plot of the resulting p-values of the t-test between CE and CB groups. No miRNAs shows adjusted p-values (FDR) below 0.1 and fold-changes above 2 (shown in red). C) Hierarchical clustering of CE and CB groups based on top 50 most variable miRNA assays. **Figure S5, Seven selected miRNAs comparing CE group with normal group.** A) Prediction probability of SVM, 53 samples with an errors = 3 (0.06>0.05). B) Area under the curve (AUC = 0.965) estimation for the microRNA panel in the CE group from the normal group.
(DOC)

## References

[1] Jemal A, Bray F, Center MM, Ferlay J, Ward E, et al (2011) Global cancer statistics. CA Cancer J Clin 61: 69–90. 21296855 10.3322/caac.20107

[2] Szabo CI, King MC (1995) Inherited breast and ovarian cancer. Hum Mol Genet 4 Spec No: 1811–1817. 10.1093/hmg/4.suppl_1.1811 8541881

[3] Chan JK, Cheung MK, Husain A, Teng NN, West D, et al (2006) Patterns and progress in ovarian cancer over 14 years. Obstet Gynecol 108: 521–528. 16946210 10.1097/01.AOG.0000231680.58221.a7

[4] Jemal A, Siegel R, Ward E, Murray T, Xu J, et al (2007) Cancer statistics, 2007. CA Cancer J Clin 57: 43–66. 17237035 10.3322/canjclin.57.1.43

[5] Siegel R, Naishadham D, Jemal A (2012) Cancer statistics, 2012. CA Cancer J Clin 62: 10–29. 22237781 10.3322/caac.20138

[6] Ahmed FY, Wiltshaw E, A'Hern RP, Nicol B, Shepherd J, et al (1996) Natural history and prognosis of untreated stage I epithelial ovarian carcinoma. J Clin Oncol 14: 2968–2975. 8918494 10.1200/JCO.1996.14.11.2968

[7] Etzioni R, Urban N, Ramsey S, McIntosh M, Schwartz S, et al (2003) The case for early detection. Nat Rev Cancer 3: 243–252. 12671663 10.1038/nrc1041

[8] Wright JD, Shah M, Mathew L, Burke WM, Culhane J, et al (2009) Fertility preservation in young women with epithelial ovarian cancer. Cancer 115: 4118–4126. 19670446 10.1002/cncr.24461

[9] Menon U, Kalsi J, Jacobs I (2012) The UKCTOCS experience—reasons for hope? Int J Gynecol Cancer 22 Suppl 1S18–20. 22543913 10.1097/IGC.0b013e318251cb47

[10] Gloss BS, Samimi G (2012) Epigenetic biomarkers in epithelial ovarian cancer. Cancer Lett. 10.1016/j.canlet.2011.12.036 22245949

[11] Bartel DP (2004) MicroRNAs: genomics, biogenesis, mechanism, and function. Cell 116: 281–297. 14744438 10.1016/s0092-8674(04)00045-5

[12] Huang Y, Shen XJ, Zou Q, Zhao QL (2010) Biological functions of microRNAs. Bioorg Khim 36: 747–752. 21317939 10.1134/s1068162010060026

[13] Mezzanzanica D, Bagnoli M, De Cecco L, Valeri B, Canevari S (2010) Role of microRNAs in ovarian cancer pathogenesis and potential clinical implications. Int J Biochem Cell Biol 42: 1262–1272. 20035894 10.1016/j.biocel.2009.12.017

[14] Kuhlmann JD, Rasch J, Wimberger P, Kasimir-Bauer S (2012) microRNA and the pathogenesis of ovarian cancer—a new horizon for molecular diagnostics and treatment? Clin Chem Lab Med 50: 601–615. 22505556 10.1515/cclm-2011-0847

[15] Sing T, Sander O, Beerenwinkel N, Lengauer T (2005) ROCR: visualizing classifier performance in R. Bioinformatics 21: 3940–3941. 16096348 10.1093/bioinformatics/bti623

[16] Wang X (2009) A PCR-based platform for microRNA expression profiling studies. RNA 15: 716–723. 19218553 10.1261/rna.1460509 PMC2661836

[17] Landgraf P, Rusu M, Sheridan R, Sewer A, Iovino N, et al (2007) A mammalian microRNA expression atlas based on small RNA library sequencing. Cell 129: 1401–1414. 17604727 10.1016/j.cell.2007.04.040 PMC2681231

[18] Hu X, Macdonald DM, Huettner PC, Feng Z, El Naqa IM, et al (2009) A miR-200 microRNA cluster as prognostic marker in advanced ovarian cancer. Gynecol Oncol 114: 457–464. 19501389 10.1016/j.ygyno.2009.05.022

[19] Liu Z, Liu J, Segura MF, Shao C, Lee P, et al (2012) MiR-182 overexpression in tumourigenesis of high-grade serous ovarian carcinoma. J Pathol 228: 204–215. 22322863 10.1002/path.4000

[20] Segura MF, Hanniford D, Menendez S, Reavie L, Zou X, et al (2009) Aberrant miR-182 expression promotes melanoma metastasis by repressing FOXO3 and microphthalmia-associated transcription factor. Proc Natl Acad Sci U S A 106: 1814–1819. 19188590 10.1073/pnas.0808263106 PMC2634798

[21] Peart TM, Correa RJ, Valdes YR, Dimattia GE, Shepherd TG (2012) BMP signalling controls the malignant potential of ascites-derived human epithelial ovarian cancer spheroids via AKT kinase activation. Clin Exp Metastasis 29: 293–313. 22249415 10.1007/s10585-011-9451-3

[22] Xu X, Dong Z, Li Y, Yang Y, Yuan Z, et al (2013) The upregulation of signal transducer and activator of transcription 5-dependent microRNA-182 and microRNA-96 promotes ovarian cancer cell proliferation by targeting forkhead box O3 upon leptin stimulation. Int J Biochem Cell Biol 45: 536–545. 23262295 10.1016/j.biocel.2012.12.010

[23] Wang YQ, Guo RD, Guo RM, Sheng W, Yin LR (2013) MicroRNA-182 promotes cell growth, invasion, and chemoresistance by targeting programmed cell death 4 (PDCD4) in human ovarian carcinomas. J Cell Biochem 114: 1464–1473. 23296900 10.1002/jcb.24488

[24] Li J, Liang S, Jin H, Xu C, Ma D, et al (2012) Tiam1, negatively regulated by miR-22, miR-183 and miR-31, is involved in migration, invasion and viability of ovarian cancer cells. Oncol Rep 27: 1835–1842. 22469921 10.3892/or.2012.1744

[25] Li XL, Hara T, Choi Y, Subramanian M, Francis P, et al (2014) A p21-ZEB1 Complex Inhibits Epithelial-Mesenchymal Transition through the MicroRNA 183-96-182 Cluster. Mol Cell Biol 34: 533–550. 24277930 10.1128/MCB.01043-13 PMC3911499

[26] Kan CW, Hahn MA, Gard GB, Maidens J, Huh JY, et al (2012) Elevated levels of circulating microRNA-200 family members correlate with serous epithelial ovarian cancer. BMC Cancer 12: 627. 23272653 10.1186/1471-2407-12-627 PMC3542279

[27] Taylor DD, Gercel-Taylor C (2008) MicroRNA signatures of tumor-derived exosomes as diagnostic biomarkers of ovarian cancer. Gynecol Oncol 110: 13–21. 18589210 10.1016/j.ygyno.2008.04.033

