# Supplementary material for: A Ten-MicroRNA Signature Identified from a Genome-Wide MicroRNA Expression Profiling in Human Epithelial Ovarian Cancer
Source: PLoS One. 2014 May 9;9(5):e96472. doi: 10.1371/journal.pone.0096472 (PMC4015980; doi:10.1371/journal.pone.0096472)

**Supporting information files S1**

**Materials & Methods**

**Materials and protocol of *Biovue’s:***

Sharpvue^TM^ miRNA First Strand Kit (*Biovue, 9000004*) for Q-PCR: *http://www.biovuetech.com/word/2011091402.pdf*

Sharpvue^TM^ 2x Universal qPCR Master Mix High Rox 50ml (*Biovue, 9000008*): *http://www.biovuetech.com/word/2011091403.pdf*

Sharpvue^TM^ Human miRNA Primer Array-A v1.0 384-well (*Biovuetech, 100002*); Sharpvue^TM^ Human miRNA Primer Array-B v1.0 384-well (*Biovuetech, 100003*); Sharpvue^TM^ Human miRNA Primer Array-C v1.0 384-well (*Biovuetech, 100004*); Sharpvue^TM^ Human miRNA Primer Array-D v1.0 384-well (*Biovuetech, 100005*); Sharpvue^TM^ Human miRNA Primer Array-E v1.0 384-well (*Biovuetech, 100006*): *http://www.biovuetech.com/word/2011091404.pdf*

**Table S1 in file S1.** Differentially expressed microRNA for EOC diagnosis between the ovarian epithelial carcinomas tissue (CE group) and ovarian normal tissue (N group).

| **Assay_Name** | **mIRID** | **logFC** | **AveExpr** | **t** | **P.Value** | **adj. P. Val** |
| --- | --- | --- | --- | --- | --- | --- |
| **hsa-miR-182** | hsa-miR-182-5p | 4.404853 | 23.81226 | 8.187034 | 4.27E-11 | 6.67E-08 |
| **hsa-miR-183** | hsa-miR-183-5p | 4.715568 | 25.19434 | 8.027705 | 7.75E-11 | 6.67E-08 |
| **hsa-miR-96** | hsa-miR-96-5p | 4.860897 | 26.69811 | 7.301426 | 1.20E-09 | 6.86E-07 |
| **hsa-miR-1271** | hsa-miR-1271-5p | -2.58333 | 25.85094 | -6.91682 | 5.11E-09 | 2.20E-06 |
| **hsa-miR-182#** | hsa-miR-182-3p | 3.475366 | 28.49623 | 6.424918 | 3.26E-08 | 1.12E-05 |
| **hsa-miR-1468** | hsa-miR-1468-5p | -2.75421 | 27.84811 | -6.36509 | 4.08E-08 | 1.17E-05 |
| **hsa-miR-135b#** | hsa-miR-135b-3p | 3.785897 | 29.81415 | 6.224998 | 6.90E-08 | 1.48E-05 |

miRID=miRID from miRBase version 20.

Log FC = log fold change

Ave. Expr = average expression

Adj. P. Val= Adjustment of P Value

**Figure S1 in file S1**.


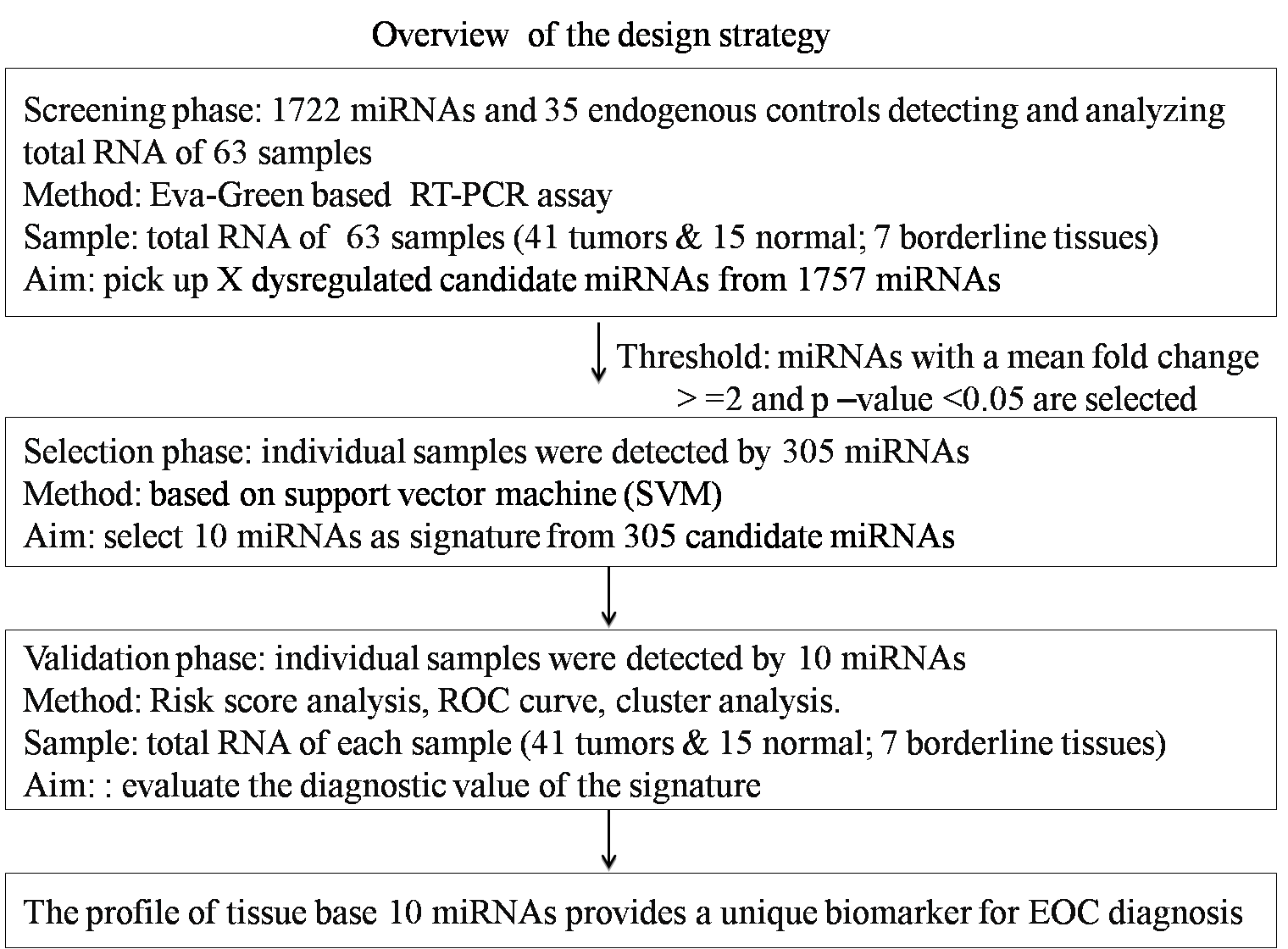


**Figure S2 in file S1.**

**
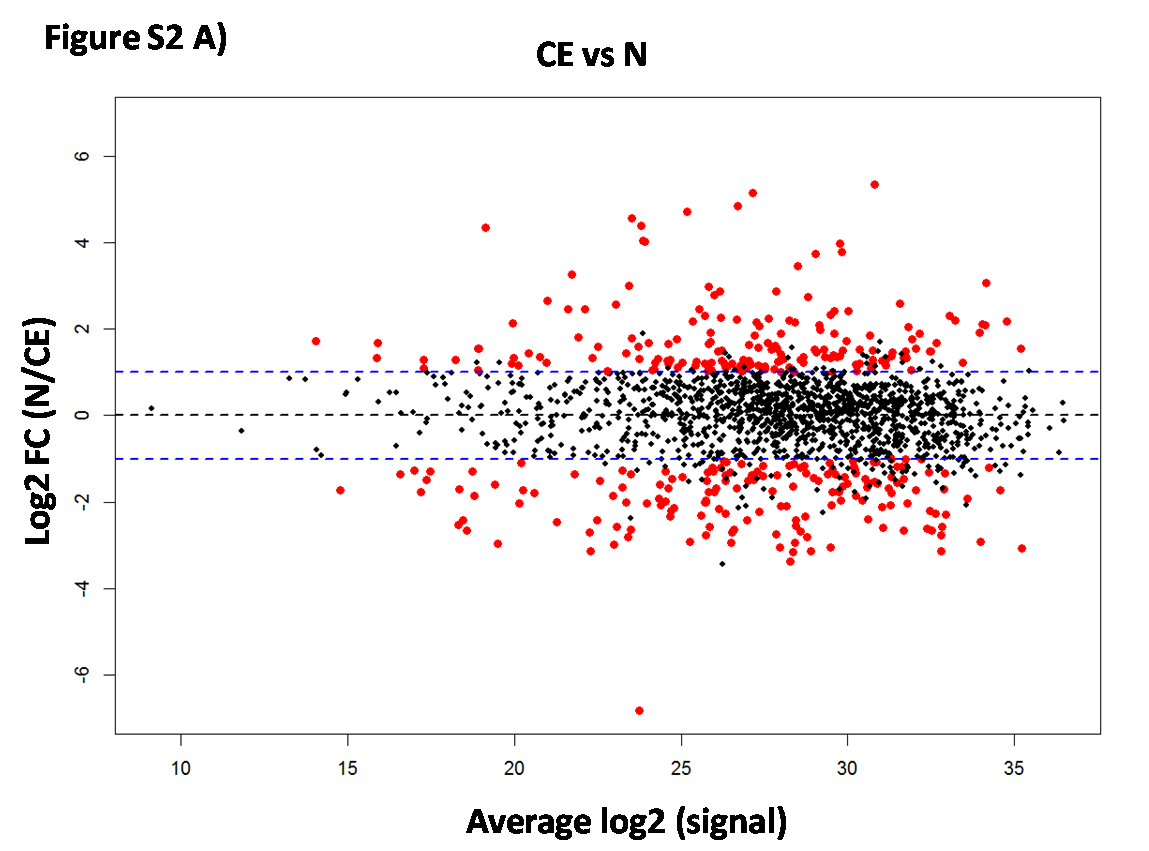
**


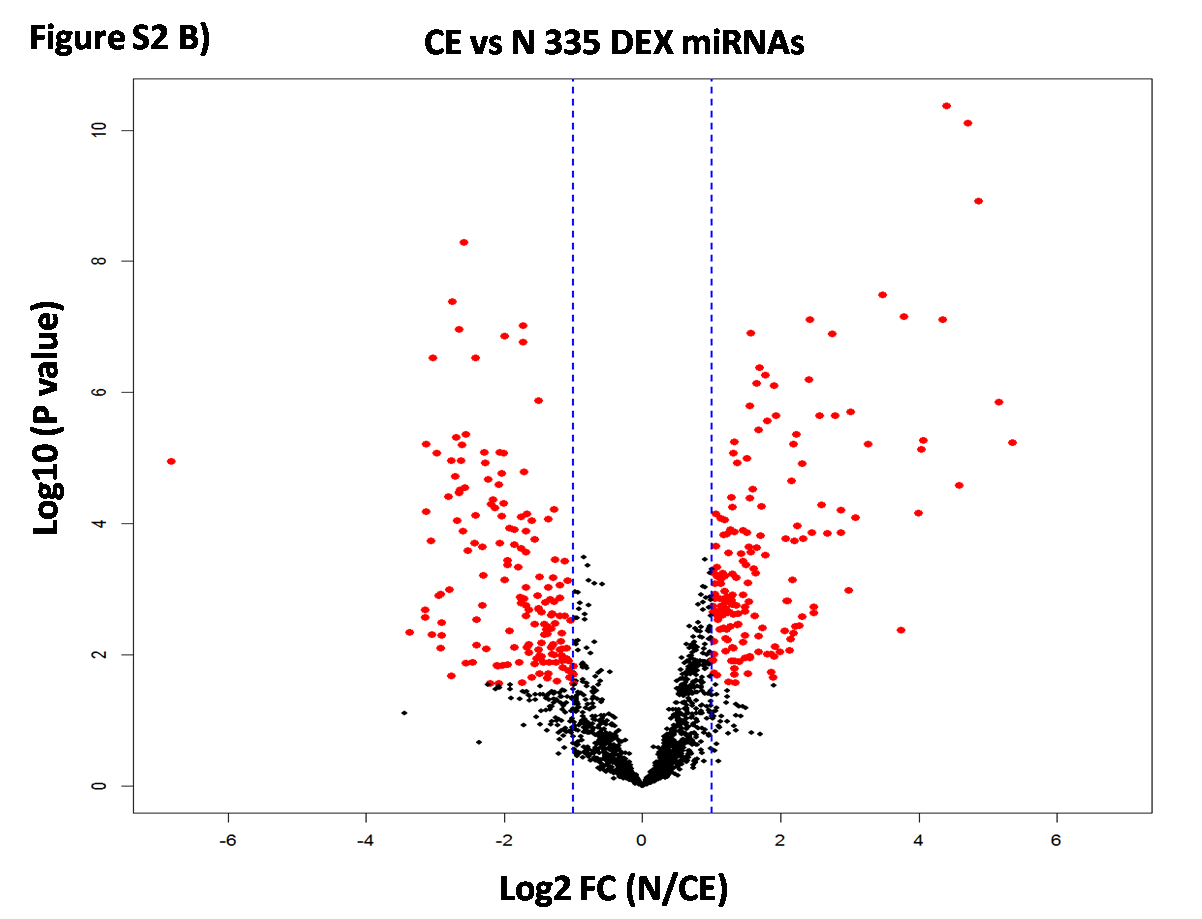


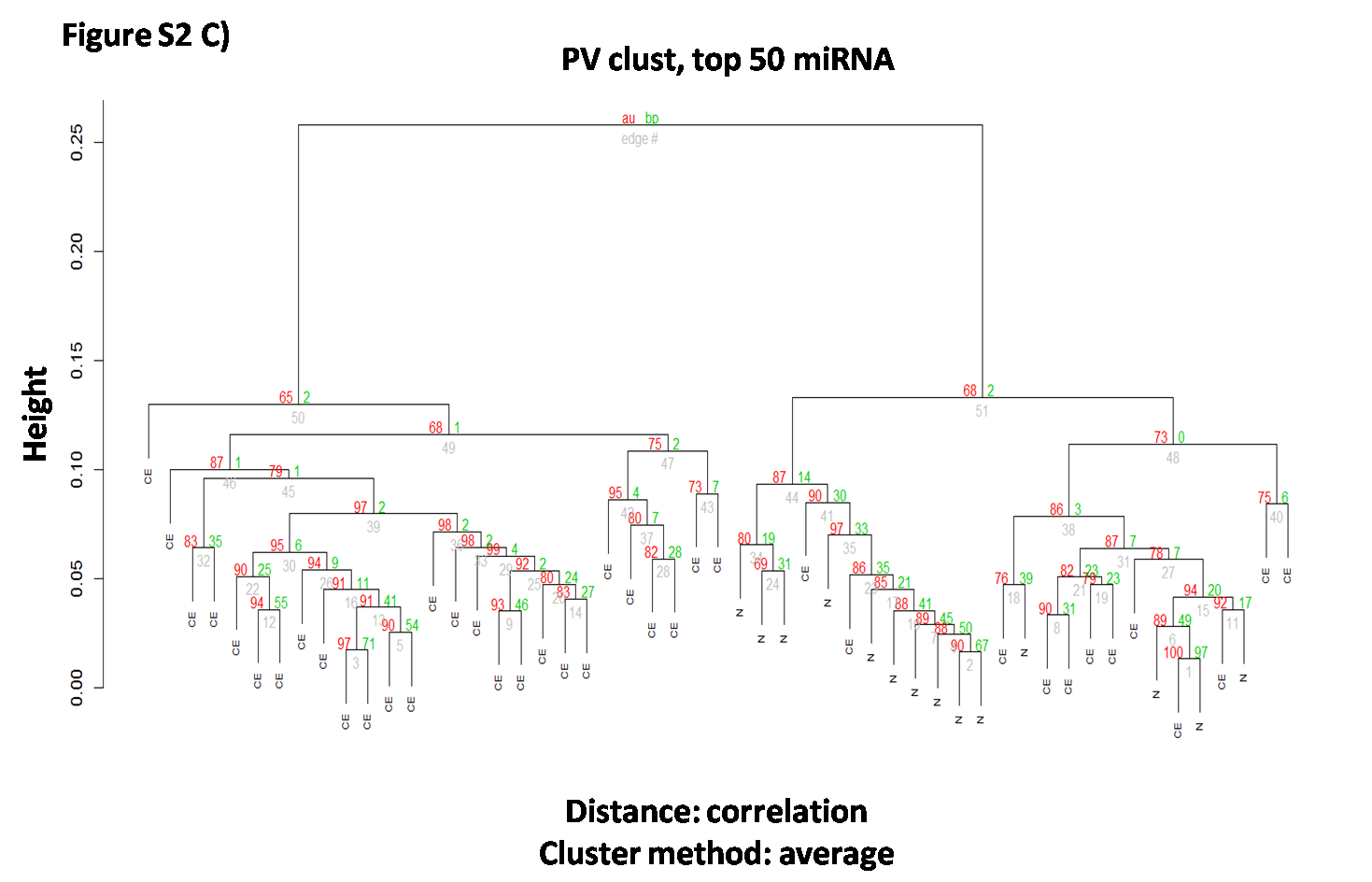


**Figure S3 in file S1.**


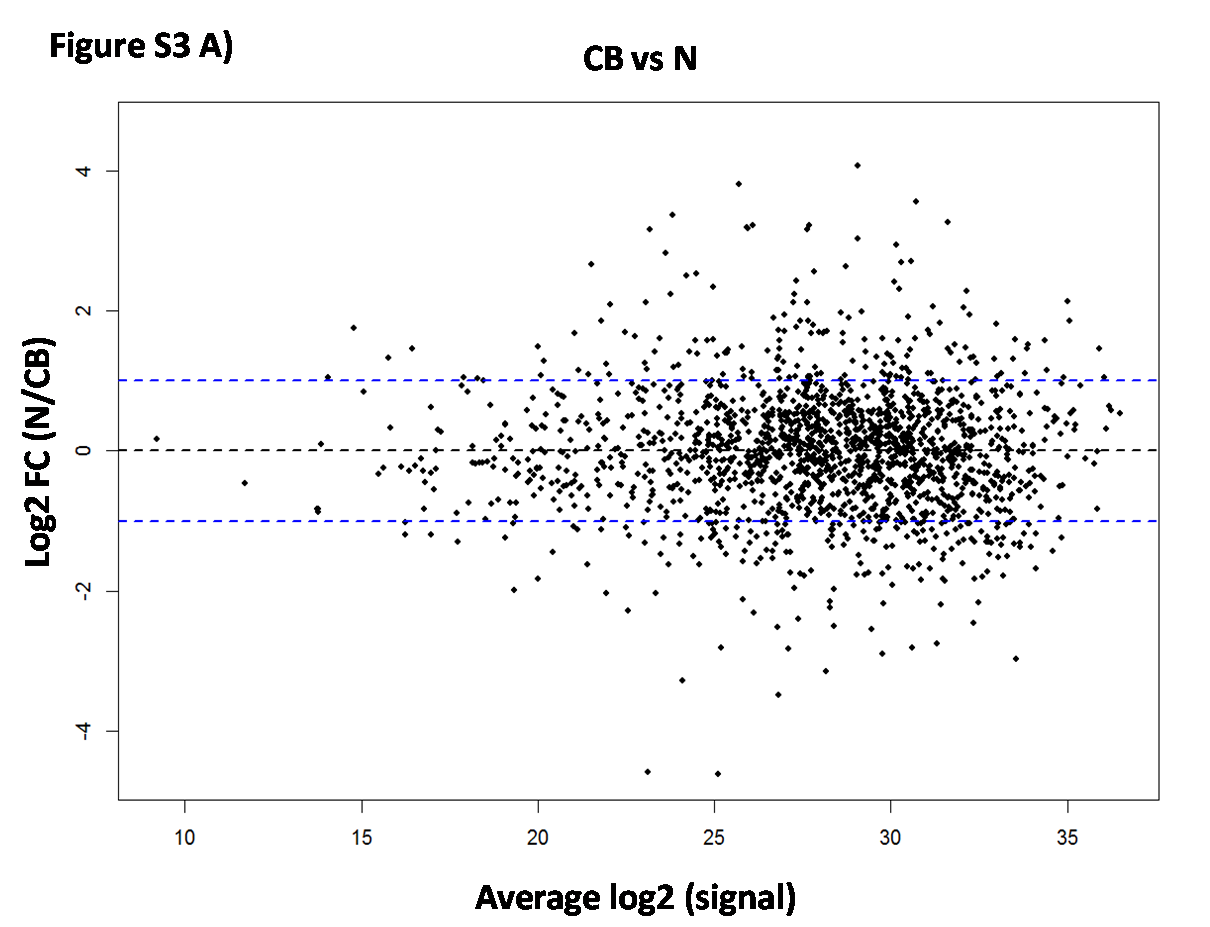


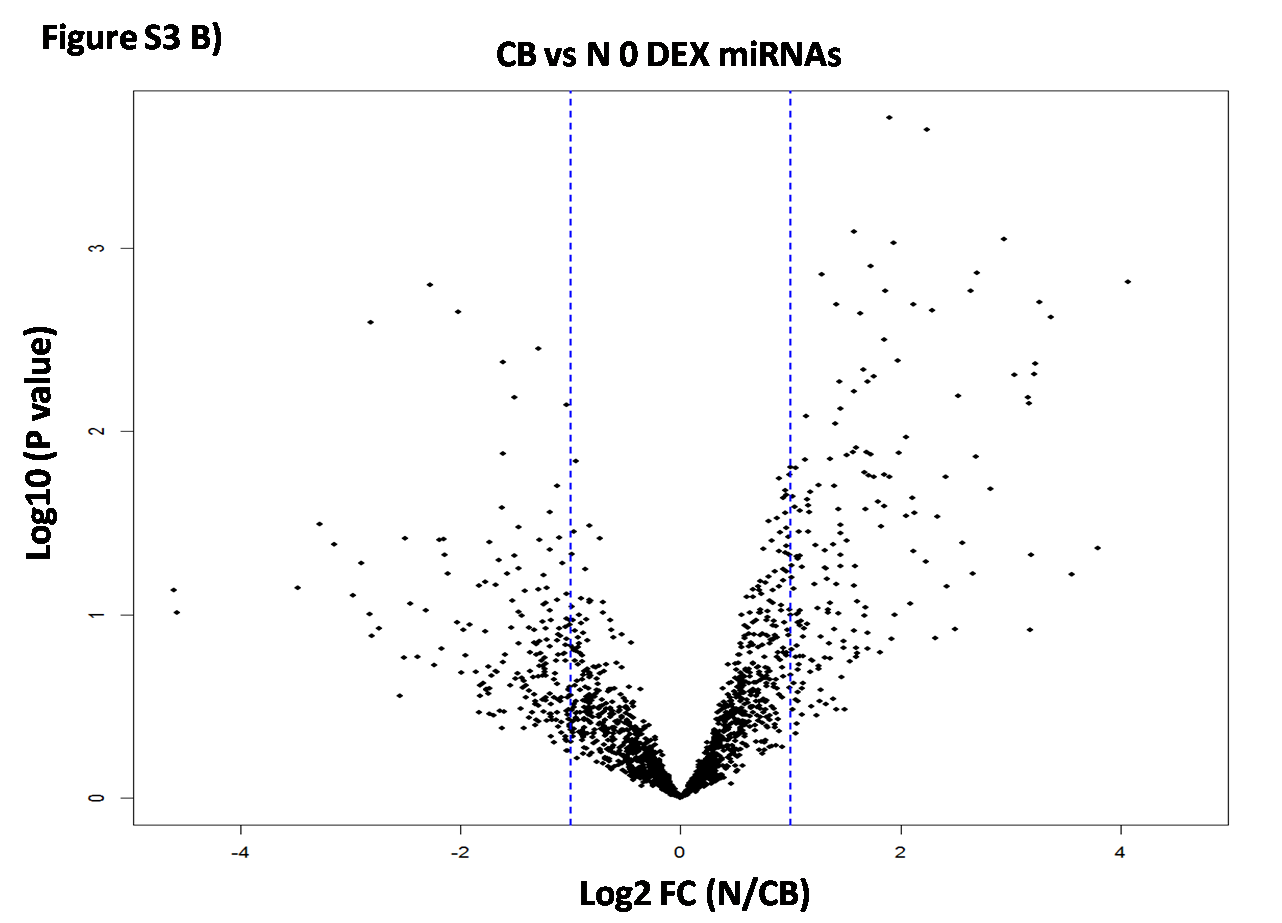


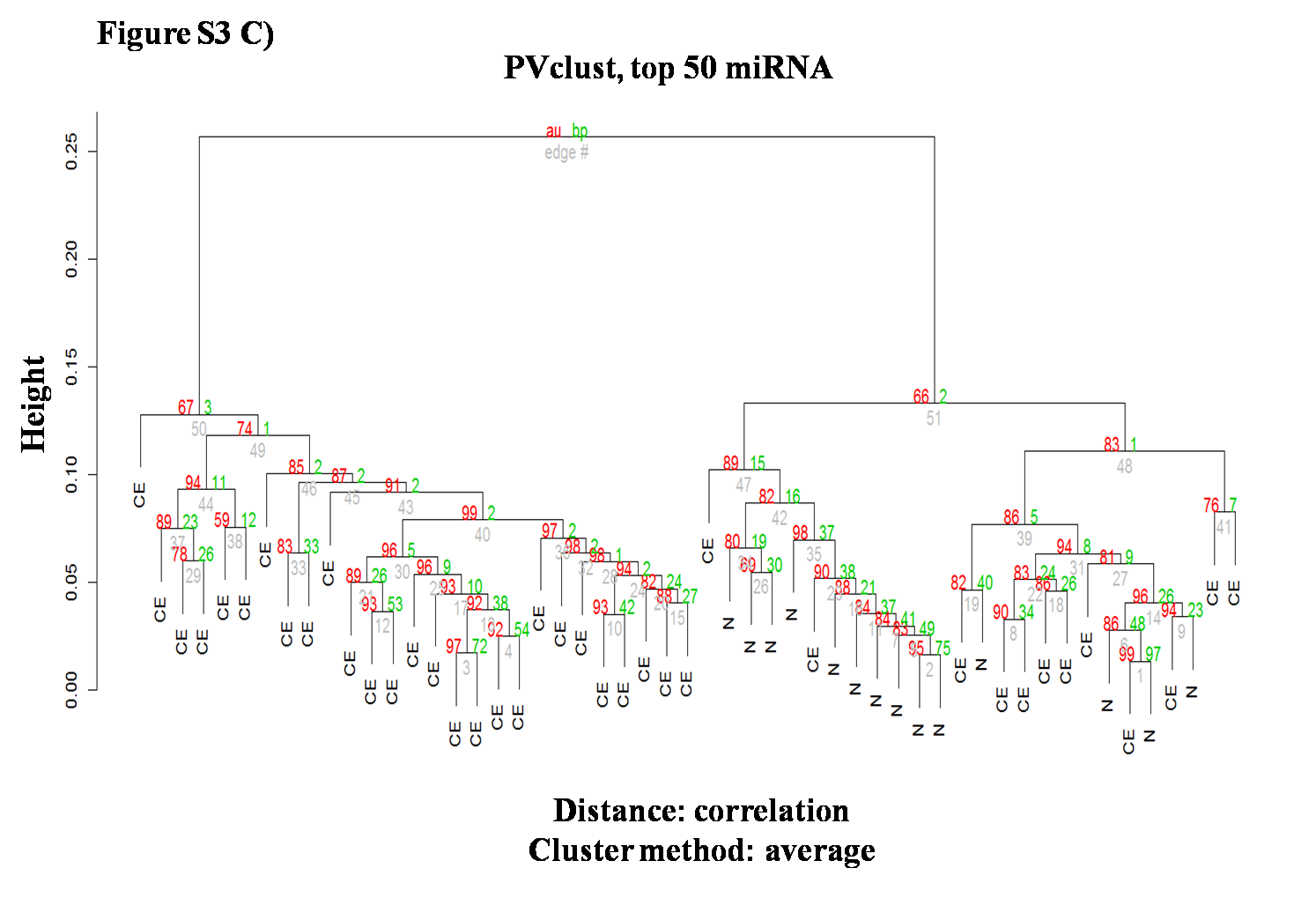


**Figure S4 in file S1.**


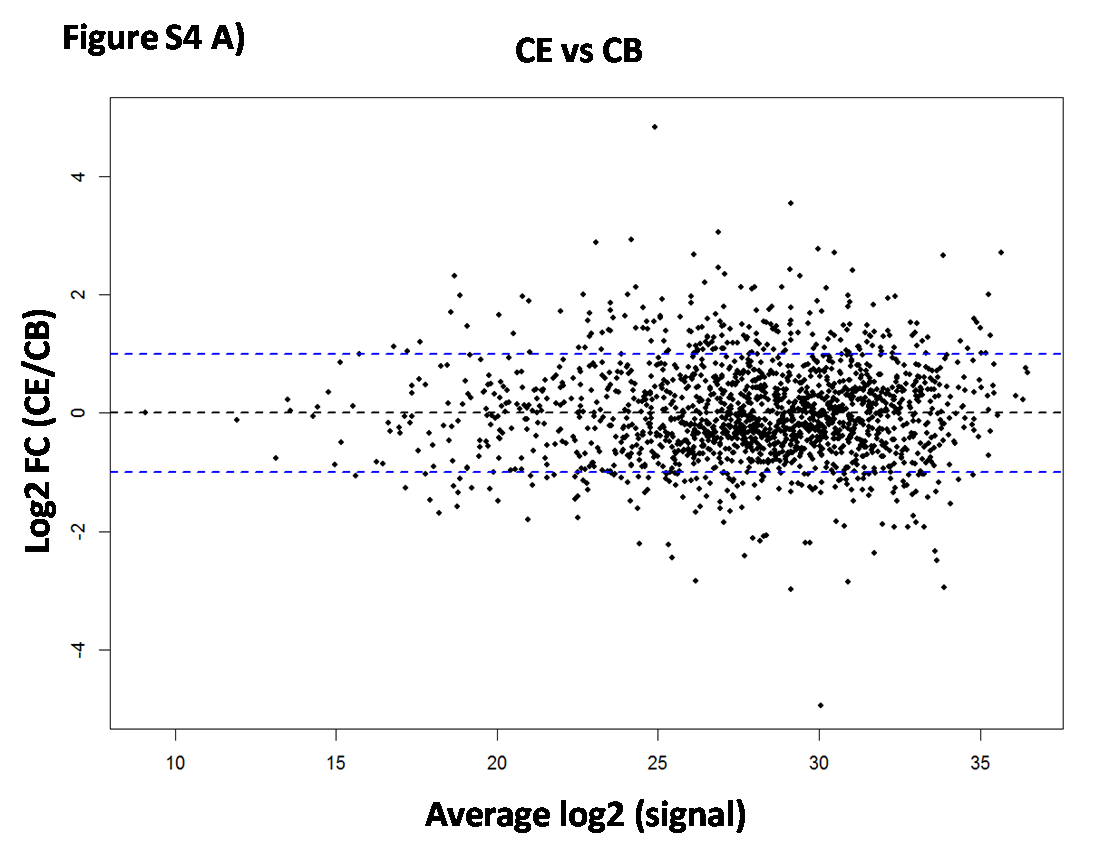


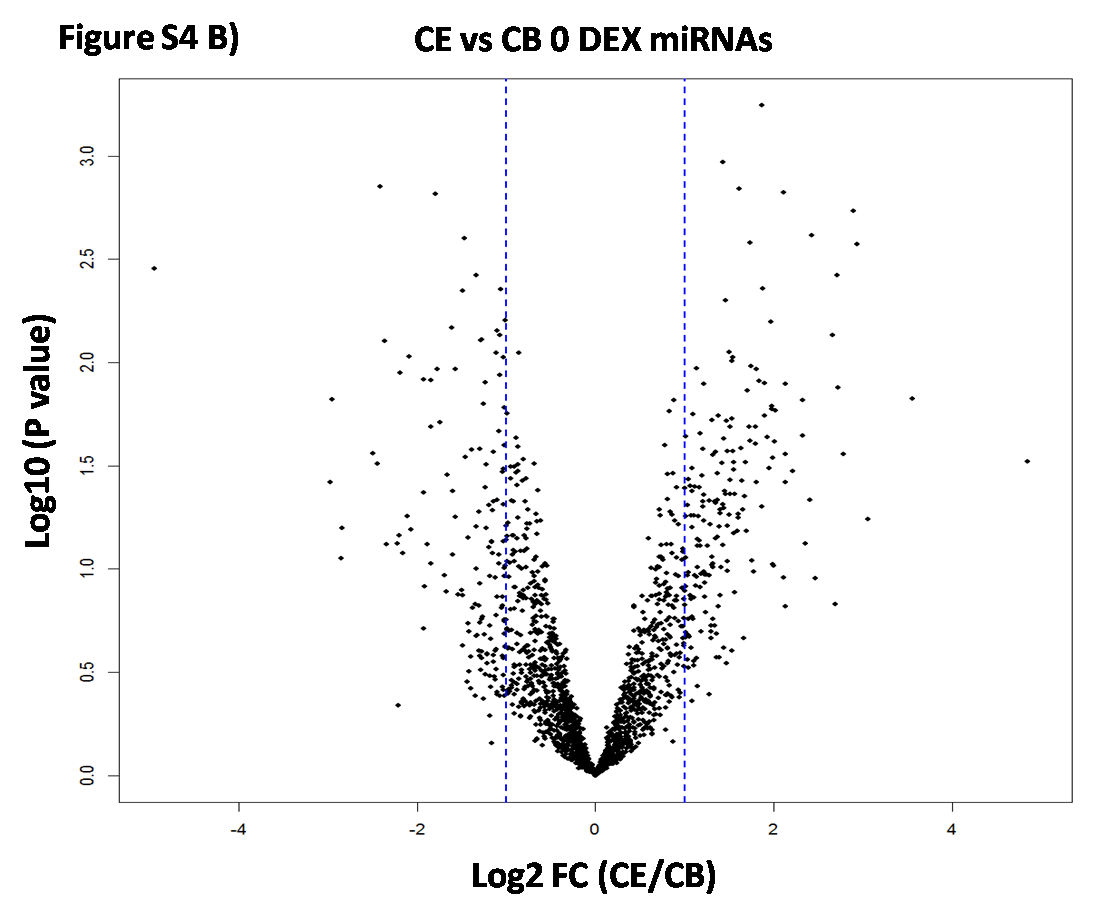


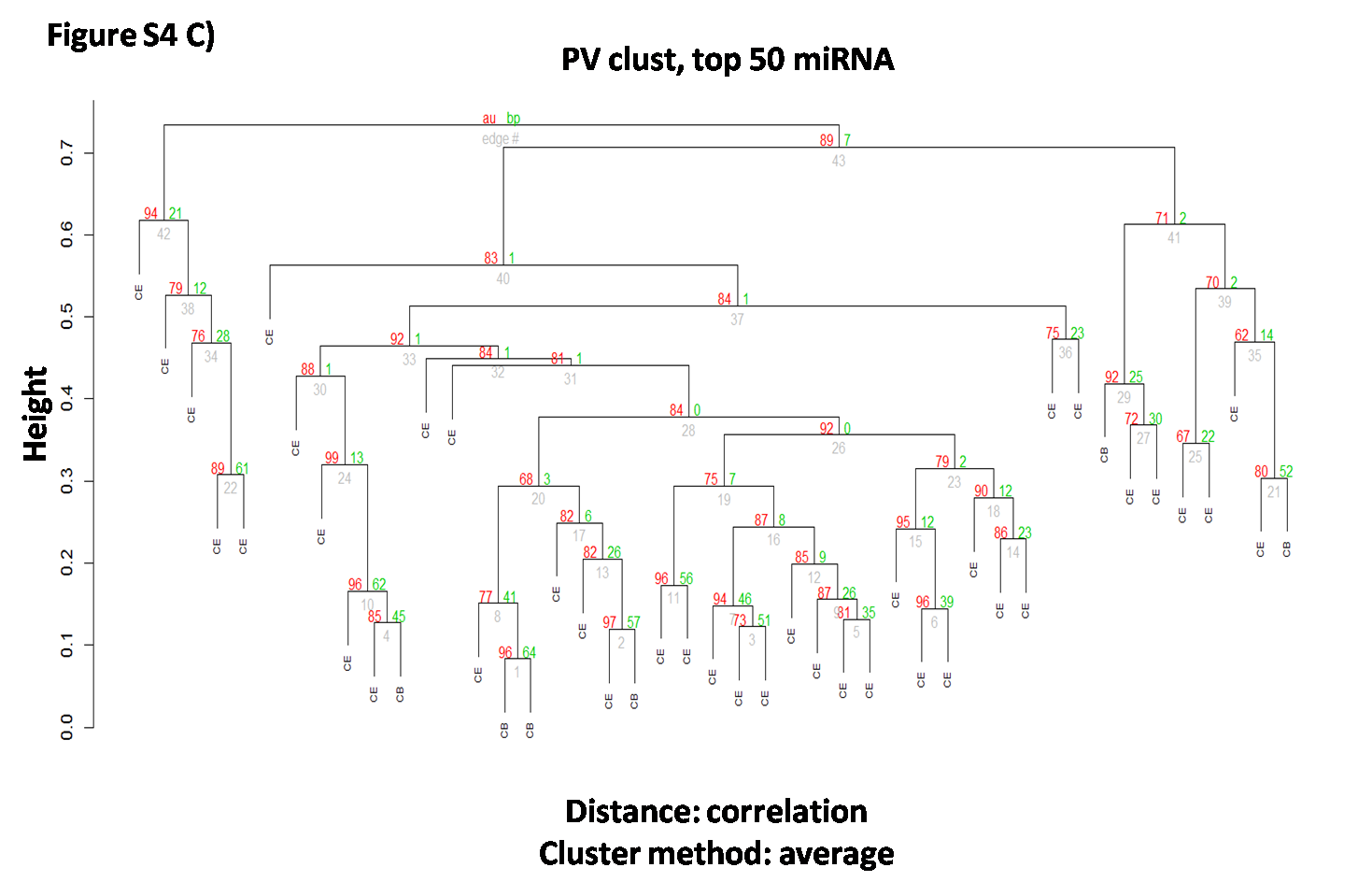


**Figure S5 in file S1.**


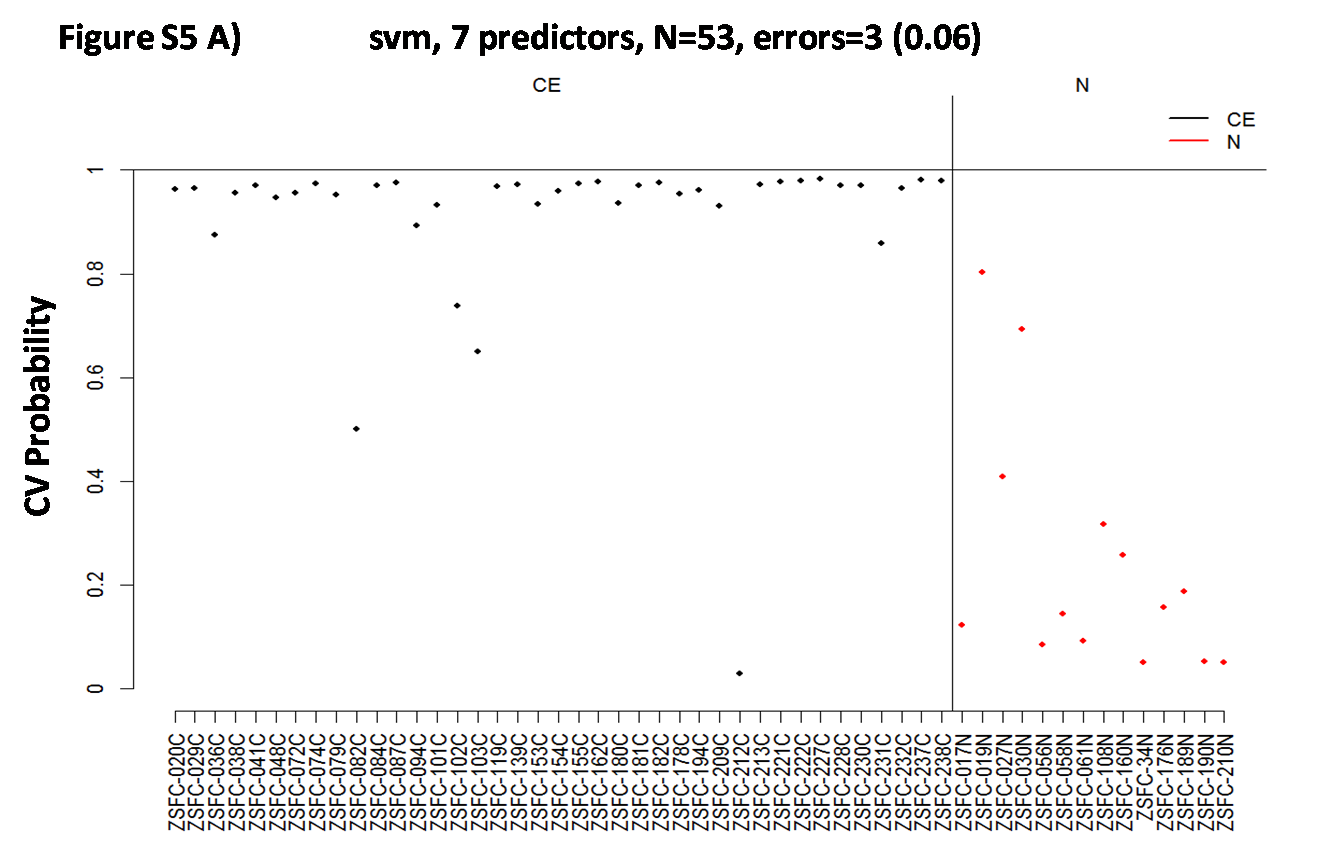


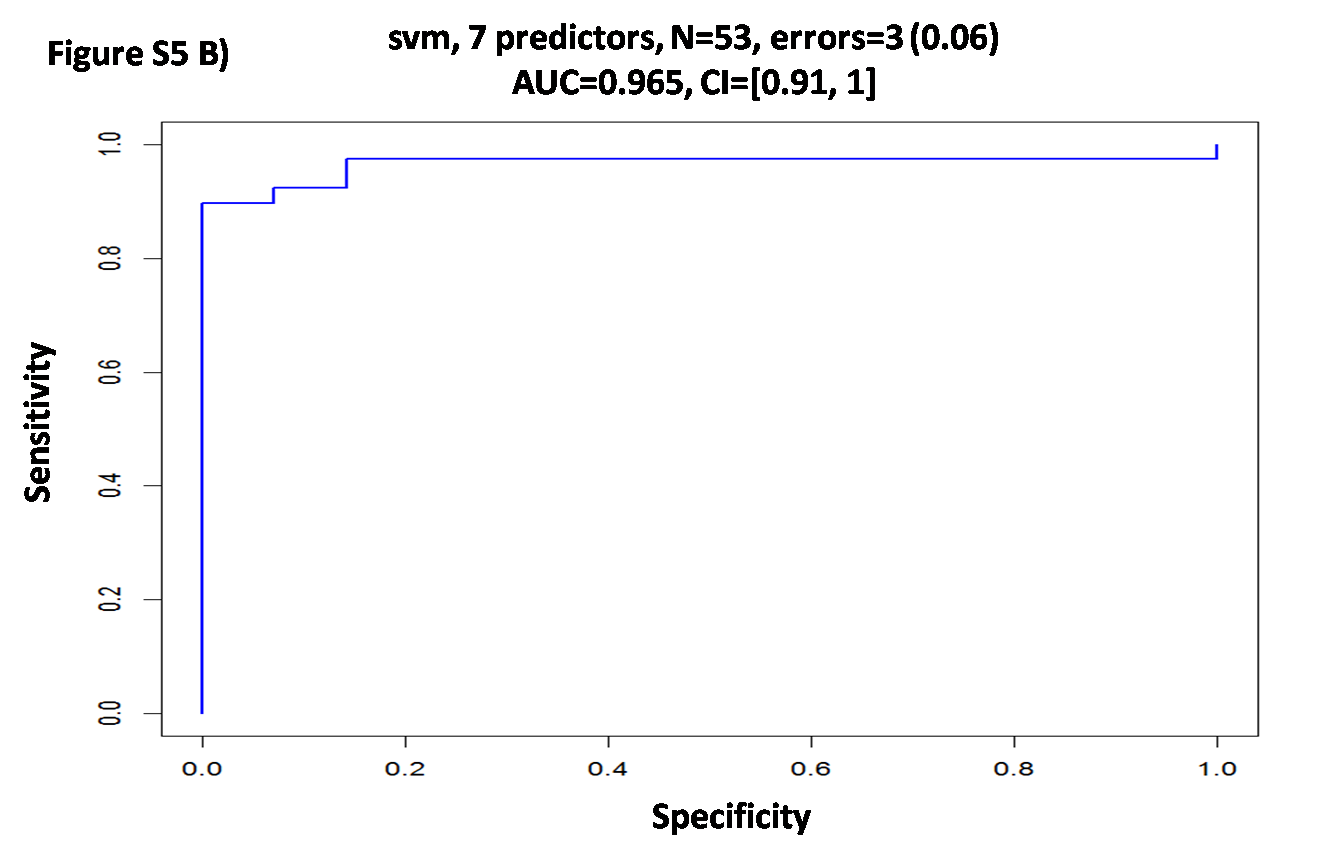

Supplement: File S1 — Supporting information figures. Figure S1, Overview of the experimental design. Figure S2, Comparison between the ovarian epithelial carcinomas tissue (CE group) and normal tissue (N group). A) MA plot of assays used to profile compared samples. B) Volcano plot of the resulting p-values of the t-test between the CE and the N groups. 335 miRNAs shows adjusted p-values (FDR) below 0.1 and fold-changes above 2 (shown in red). C) Hierarchical clustering of CE and N groups based on top 50 most variable miRNA assays. Figure S3, Comparison between the ovarian borderline tissue (CB group) and normal tissue (N group). A) MA plot of assays used to profile compared samples. B) Volcano plot of the resulting p-values of the t-test between the CB and the N groups. No miRNA shows adjusted p-values (FDR) below 0.1 and fold-changes above 2 (shown in red). C) Hierarchical clustering of CB and N group based on top 50 most variable miRNA assays. Figure S4, Comparison between the ovarian epithelial carcinomas tissue (CE group) and ovarian borderline tissue (CB group). A) MA plot of assays used to profile compared samples. B) Volcano plot of the resulting p-values of the t-test between CE and CB groups. No miRNAs shows adjusted p-values (FDR) below 0.1 and fold-changes above 2 (shown in red). C) Hierarchical clustering of CE and CB groups based on top 50 most variable miRNA assays. Figure S5, Seven selected miRNAs comparing CE group with normal group. A) Prediction probability of SVM, 53 samples with an errors = 3 (0.06>0.05). B) Area under the curve (AUC = 0.965) estimation for the microRNA panel in the CE group from the normal group. (DOC) [file pone.0096472.s001.doc]
